# Antiherpetic Activity of a Root Exudate from *Solanum lycopersicum*

**DOI:** 10.3390/microorganisms12020373

**Published:** 2024-02-11

**Authors:** Greta Bajetto, Davide Arnodo, Matteo Biolatti, Linda Trifirò, Camilla Albano, Selina Pasquero, Francesca Gugliesi, Eva Campo, Francesca Spyrakis, Cristina Prandi, Marco De Andrea, Valentina Dell’Oste, Ivan Visentin, Marco Blangetti

**Affiliations:** 1 Department of Public Health and Pediatric Sciences, University of Turin, 10126 Turin, Italy; greta.bajetto@unito.it (G.B.); matteo.biolatti@unito.it (M.B.); linda.trifiro@unito.it (L.T.); camilla.albano@unito.it (C.A.); selina.pasquero@unito.it (S.P.); francesca.gugliesi@unito.it (F.G.); marco.deandrea@unito.it (M.D.A.); 2 Center for Translational Research on Autoimmune and Allergic Disease (CAAD), 28100 Novara, Italy; 3 Department of Chemistry, University of Turin, 10125 Turin, Italy; davide.arnodo@unito.it (D.A.); cristina.prandi@unito.it (C.P.); 4 Department of Agricultural, Forestry, and Food Sciences, University of Turin, 10095 Turin, Italy; eva.campo@unito.it (E.C.); ivan.visentin@unito.it (I.V.); 5 Department of Drug Science and Technology, University of Turin, 10125 Turin, Italy; francesca.spyrakis@unito.it

**Keywords:** antivirals, herpesviruses, natural compounds, tomatoes, plant specialized metabolites, rhizosphere

## Abstract

The rise of drug resistance to antivirals poses a significant global concern for public health; therefore, there is a pressing need to identify novel compounds that can effectively counteract strains resistant to current antiviral treatments. In light of this, researchers have been exploring new approaches, including the investigation of natural compounds as alternative sources for developing potent antiviral therapies. Thus, this work aimed to evaluate the antiviral properties of the organic-soluble fraction of a root exudate derived from the tomato plant *Solanum lycopersicum* in the context of herpesvirus infections. Our findings demonstrated that a root exudate from *Solanum lycopersicum* exhibits remarkable efficacy against prominent members of the family *Herpesviridae*, specifically herpes simplex virus type 1 (HSV-1) (EC_50_ 25.57 µg/mL, SI > 15.64) and human cytomegalovirus (HCMV) (EC_50_ 9.17 µg/mL, SI 32.28) by inhibiting a molecular event during the herpesvirus replication phase. Moreover, the phytochemical fingerprint of the *Solanum lycopersicum* root exudate was characterized through mass spectrometry. Overall, these data have unveiled a novel natural product with antiherpetic activity, presenting a promising and valuable alternative to existing drugs.

## 1. Introduction

Herpesviruses pose a significant threat to human health, causing chronic and recurring infections that profoundly impact the quality of life for affected individuals. Notable members of this viral family include herpes simplex virus type 1 (HSV-1) and human cytomegalovirus (HCMV) [1,2]. Many members of the *Herpesviridae* family exhibit high seroprevalence globally; HCMV reaches more than 50% of the general population, and HSV, more than 60%. Around 500 million people worldwide, aged 15 to 50, have genital infections caused by HSV-1 or HSV-2, while the incidence of cold sores is second globally only to the common cold. HCMV infection stands as the prevailing congenital infection globally, with an estimated occurrence in developed nations ranging from 0.6% to 0.7% of all live births. This translates to around 60,000 neonates born annually with HCMV infection in the Western world. Notably, in developing countries, the prevalence is even more pronounced, affecting between 1% and 5% of all live births [2,3,4]. The efficacy of existing treatments is compromised by resistant strains of herpesviruses, highlighting the pressing need to explore alternative compounds and formulations [5,6,7]. Therefore, the quest for new antiherpetic drugs is a critical global challenge not only to alleviate the suffering caused by herpesvirus, but also to counter drug-resistant herpesvirus strains, improve treatment outcomes, and tackle the evolving challenges exacerbated by the emergence of viral escape mutants.

Within this framework, herbal medicines and purified natural products derived from plants, marine organisms, and other natural sources have garnered considerable attention as a rich resource for the development of innovative antiviral drugs. This includes a diverse array of substances, including essential oils, plant extracts, small peptides from animal sources, bacteriocins, and several categories of plant compounds (such as alkaloids, triterpenoids, flavonoids, and phenols) renowned for their antimicrobial and antiviral properties [8,9,10]. The use of natural compounds as antivirals presents several advantages over synthetic drugs. Indeed, they are often derived from renewable sources, making them more sustainable and environmentally friendly. Moreover, these compounds may have fewer adverse side effects compared to synthetic drugs, as they have evolved alongside living organisms and are more compatible with human biology [11,12].

*Solanum lycopersicum*, commonly referred to as the tomato plant, is a species of flowering plant that belongs to the *Solanaceae* family. It ranks as the second most widely cultivated and consumed vegetable worldwide, making it both an essential model organism for plant research and a significant commercial crop. In addition to its noteworthy nutritional value, *Solanum lycopersicum* has been studied for its potential medicinal properties which have been recognized and utilized for centuries [13,14,15,16]. Notably, tomato extracts contain a diverse array of bioactive compounds, such as flavonoids and carotenoids. These specialized metabolites accumulate in various tissues of the tomato plant and are released as root exudates and volatiles [17]. Tomato root exudates are rich in organic acids, sugars, and amino acids. Among these, glucose and fructose stand out as the primary sugars; malic, citric, and succinic acids are the predominant organic acids [18]; and glutamic acid, aspartic acid, leucine, isoleucine, and lysine are the main amino acids [19]. The secretion levels differ based on the growth phase of the tomato and the presence of particular microorganisms [20].

These compounds impart a significant nutraceutical value to tomatoes, positioning them as functional foods that can positively impact various pathological conditions [16]. One notable example is their ability to reduce the production of pro-inflammatory molecules, thereby mitigating inflammation and potentially avoiding the onset of chronic inflammatory diseases [21]. Furthermore, tomatoes have also garnered attention for their potential cardiovascular benefits. The abundant potassium content found in *Solanum lycopersicum* assists in regulating blood pressure by counteracting the effects of sodium, thus supporting a healthy cardiovascular system. Lastly, in terms of medicinal properties, it is suggested that it may contribute to a reduction in cancer risks, particularly for lung and prostate cancers [22,23].

Despite numerous investigations into the therapeutic properties of tomatoes, there is a lack of studies exploring their potential antiviral effects, particularly those of root exudates. Interestingly, the antiviral efficacy of tomatidine, a steroidal alkaloid obtained from the stem and leaves of unripe, green tomatoes, has been demonstrated against all four dengue virus (DENV) serotypes and Zika virus (ZIKV) in the Huh7 in vitro model. It disrupts various stages of DENV replication, especially post-virus–cell binding and internalization. The cellular activating transcription factor 4 (ATF4) may enhance the antiviral effect, yet it is not entirely responsible for it. Notably, no antiviral activity was detected for West Nile virus (WNV), another mosquito-borne flavivirus [24]. Tomatidine and its structural derivatives solasodine and sarsasapogenin were also effective against multiple chikungunya virus (CHIKV) strains, primarily during the early stages of infection [25]. In the context of DNA viruses, tomatine displayed inhibitory activity against HSV-1, possibly through the interaction of the sugar moiety of the glycosidic chain with the viral envelope [26].

In this study, recognizing the need to continue searching for novel antiviral agents, we aimed to investigate the in vitro antiviral activity of a root exudate derived from *Solanum lycopersicum* against two members of the herpesvirus family (specifically HSV-1 and HCMV). Additionally, the phytochemical fingerprint of the exudate was determined by ESI-MS analyses, disclosing the occurrence of several bioactive compounds (carotenoids, phytosterols, and polyphenols) which might play a synergic role in determining the antiviral activity of the tomato root exudate. In summary, these data highlight *Solanum lycopersicum* as a natural product with the potential to serve as a valuable alternative to existing antiviral drugs.

## 2. Materials and Methods

### 2.1. Plant Material and Tomato Extract Preparation

**Figure 1:**
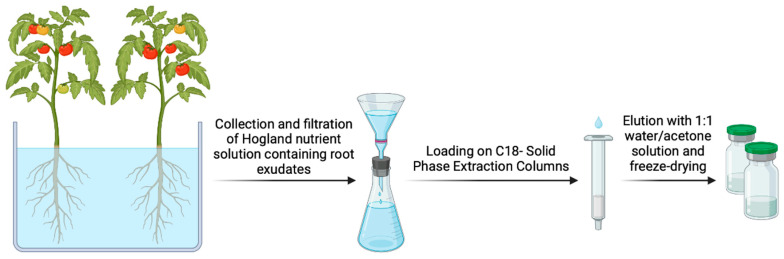
Strategy for the preparation of extracts from tomato root exudates (created with BioRender).

Tomato (*Solanum lycopersicum* cv. M82) seeds were surfaced-sterilized in 4% sodium hypochlorite containing 0.02% (*v*/*v*) Tween 20, washed thoroughly with sterile water, and germinated for 2 days in a plate on moistened filter paper at 25 °C in the darkness. Subsequently, 4-week-old plants were transferred in an aeroponic system and grown with Hogland nutrient solution (0.2 μM ZnSO_4_, 0.2 μM CuSO_4_, 1 mM Ca(NO_3_)_2_, 80 μM KH_2_PO_4_, 250 μM KNO_3_, 20 μM FeNa EDTA, 1.8 μM MnCl_2_, 0.2 μM Co(NO_3_)_2_, 9 μM H_3_BO_3_, 0.2 μM NiSO_4_, 1 mM MgSO_4_, pH 6.0). The plants were cultured in a growth chamber set at the following parameters: 16/8 h day/night cycle, 25 °C, 65% humidity, and 200 µmol s^–1^ m^–2^ of photosynthetic photon flux density (PPFD). The nutrient solution was renewed and alternated with a version containing an equimolar quantity of KCl instead of KH_2_PO_4_. The nutrient solution containing the root exudates was collected and filtered twice a week by a 0.2 mm sieve. Subsequently, the root exudate was loaded onto the preequilibrated column (Grace Pure C18-Fast 500 mg/3 mL SPE) and eluted with 5 mL of 1:1 water/acetone solution. Finally, the eluted solution was dried with a rotary evaporator and stocked at −20 °C (Figure 1) [27]. At the time of use, 10 mg of lyophilized root exudate was dissolved in 1 mL of DMSO/H_2_O mixture (1:1) and subsequently diluted in the culture medium.

### 2.2. Phytochemical Analysis

Acetonitrile (HPLC-grade) and formic acid (mass-spectrometry-grade, 98%) were purchased from Sigma Aldrich. The tomato root exudate sample was diluted in acetonitrile (1.0 mg/mL) with 0.1% formic acid as a charge carrier.

Direct-infusion positive ESI-MS analysis was performed on a 3200 QTRAP^®^ LC-MS/MS System (AB Sciex) equipped with a syringe pump. The sample was infused at a flow rate of 10 µL/min. The paramete, rs adopted for the ESI source were as follows: source voltage 5.0 kV; heated capillary temperature 350 °C; N_2_ curtain gas flow rate 20 (arbitrary units); declustering potential 20 V; entrance potential 10 V. Total ion current (TIC) mode was used to record the abundances of the ionized adducts. In the full-scan mode, masses were scanned as centroid data from *m*/*z* 100 to 700 at a rate of 1 scan/s (averaged over 5 min). Data acquisition and processing and instrumental control were performed using the Analyst^®®^ software package version 1.7 (AB Sciex).

### 2.3. Cells and Viruses

African green monkey kidney cells (VERO, ATCC CCL-81™) and primary human foreskin fibroblasts (HFFs, ATCC SCRC-1041™) were propagated in Dulbecco’s Modified Eagle Medium (DMEM; Sigma-Aldrich, Milan, Italy) complemented with 1% streptomycin/penicillin solution (Sigma-Aldrich) and 10% fetal bovine serum (heat-inactivated) (Sigma-Aldrich).

The clinical isolate of HSV-1 (obtained from Valeria Ghisetti, “Amedeo di Savoia” Hospital, Turin, Italy) and the AD169 HCMV strain (ATCC VR538) were grown and titrated by a standard plaque assay on VERO and HFF cells, respectively, on a 96-well plate (~30,000 cells/well), as previously described [28,29].

### 2.4. Cytotoxicity Assay

VERO and HFF cells were seeded in a 96-well culture plate (~30,000 cells/well) and exposed to increasing concentrations of either tomato extract or vehicle control (DMSO/H_2_O mixture 1:1). After 48 h (for VERO) or 144 h (for HFF) of incubation, cell viability was determined using the 3-(4,5-dimethylthiazol-2-yl)-2,5-diphenyltetrazolium bromide (MTT) (Sigma-Aldrich) assay, as previously described [30].

### 2.5. Virus Yield Reduction Assay

VERO and HFF cells were plated in 24-well plates (~200,000 cells/well) and pre-treated with different concentrations of tomato extracts, vehicle, or the reference drugs Acyclovir (ACV, Sigma-Aldrich) for HSV-1 and Ganciclovir (GCV, Sigma-Aldrich) for HCMV for 1 h at 37 °C. They were then infected with HSV-1 (VERO) or HCMV (HFF) at an MOI of 0.1 plaque-forming units per cell (PFU/cell) in the presence of treatments. Following virus adsorption (2 h at 37 °C), the viral inoculum was removed, and the cultures were maintained in a medium that contained the extract for 48 h (HSV-1) or 144 h (HCMV). Cells and supernatants (combined) were then collected and lysed using three freeze (liquid nitrogen)/thaw (37 °C) cycles. Virus replication was assessed by titrating the infectivity of the samples obtained from the virus yield reduction assay with a standard plaque assay on VERO (HSV-1) and HFF (HCMV) previously plated in a 96-well plate (~30,000 cells/well). After 48 h (HSV-1) or 144 h (HCMV), cells were fixed and stained with a crystal violet solution (0.1% crystal violet, 20% ethanol). Plaques were microscopically counted, and the mean plaque counts for each drug concentration were expressed as PFU/mL. The concentration that produced a 50% reduction in plaque formation (EC_50_) was determined for each test by nonlinear regression (curve fitting analysis) in GraphPad Prism software version 8.02 for Windows. The selectivity index (SI) reflects the CC_50_-to-EC_50_ ratio.

### 2.6. Time of Addition Assay

VERO and HFF cells were seeded in 24-well plates (~200,000 cells/well) and treated with various concentrations of tomato extract or vehicle solution at different time points: (i) 1 h pre-infection as pre-treatment, (ii) only during the 2 h of viral adsorption, (iii) post-infection until sample collection. In all conditions, cells were infected with HSV-1 or HCMV (MOI 0.1 PFU/cell). After 48 h (VERO) or 144 h (HFF), cells and supernatants were collected and disrupted using three freeze (liquid nitrogen)/thaw (37 °C) cycles. The level of virus replication was then assessed by titrating the infectivity of the samples by a plaque assay as described in the virus yield reduction assay section.

### 2.7. Western Blot Analysis

VERO and HFF cells were seeded in 6-well plates (~10^6^ cells/well), treated as described in the virus yield reduction assay section, and infected at an MOI of 1. Cells were collected at different time points, and whole-cell protein extracts were prepared using Radioimmunoprecipitation Assay (RIPA) buffer. Then, 30 µg of protein extracts were subjected to electrophoresis on sodium dodecyl sulfate–polyacrylamide (SDS) gels and transferred to Immobilon-P membranes (Biorad). Membranes were blocked with 5% nonfat dry milk in TBS-Tween 0.05% and incubated overnight at 4 °C with the primary antibodies. The following mouse monoclonal primary antibodies were used: anti-HSV-1 ICP4 (clone 10F1, H1A021-100, Virusys Corporation, Taneytown, MD, USA), anti-HSV-1/2 gD (clone 2C10, HA025-100, Virusys Corporation), anti-HCMV IEA (clone CH160, P1215, Virusys Corporation), pp65 (clone 3A12, CA003-100, Virusys Corporation), pp28 (clone 5C3, CA004-100, Virusys Corporation), anti-Actin (clone C4, MAB1501, Sigma-Aldrich) (all at 1:1000 dilution in 5% nonfat dry milk, TBS-Tween 0.05%). After washing with TBST buffer (500 mM NaCl, 20 mM Tris pH 7.4, 0.05% Tween 20), the membranes were incubated with an HRP-conjugated anti-mouse secondary antibody (1:3000 diluted, Sigma-Aldrich) for 2 h at room temperature, and immunocomplexes were visualized using an enhanced chemiluminescence detection kit (SuperSignal West Pico Chemiluminescent Substrate, Thermo SCIENTIFIC, Waltham, MA, USA).

### 2.8. DNA Extraction and Viral Load

VERO and HFF cells were seeded in 24-well plates (~200,000 cells/well), treated as described in the virus yield reduction assay section, and infected at an MOI of 1. After 24 (HSV-1) or 72 (HCMV) hours, cells were collected, and the cellular-associated DNA was isolated using the TRI Reagent solution (Sigma-Aldrich) according to the manufacturer’s instructions. The extracted viral DNA was quantified by quantitative real-time PCR (qPCR) analysis using a CFX Touch Real-Time PCR Detection System (BioRad, Hercules, CA, US). The viral DNA copy numbers were quantified using primers to amplify a segment of the IE1 gene for HCMV (Fw, 5′-TCAGTGCTCCCCTGATGAGA-3′; Rv, 5′-GATCAATGTGCGTGAGCACC-3′) or gE for HSV (Fw, 5′-TGTCTGTATCAGCCGCAGC-3′; Rv, 5′-TTCTGGAACACCCCGCGTA-3′). Intracellular viral DNA copy numbers were normalized to GAPDH (Fw, 5′-AGTGGGTGTCGCTGTTGAAGT-3′; Rv, 5′-AACGTGTCAGTGGTGGACCTG-3′). To create a standard curve for each analysis, genomic DNA mixed with a gE2- or IE1-encoding plasmid (pAcUW51-CgE, a gift from Pamela Bjorkman; Addgene plasmid #13762, http://n2t.net/addgene:13762 accessed on 31 December 2023, RRID: Addgene_13762 [31] (Addgene, Watertown, MA, USA; pSG-IE72, available at the Department of Public Health and Pediatric Sciences, Turin, Italy) was serially diluted and analyzed in parallel.

### 2.9. Statistical Analysis

The statistical tests were performed using GraphPad Prism version 8.0.2 for Windows (GraphPad, Boston, MA, USA. Data were presented as the mean value and standard error of the mean (SEM). Differences were considered statistically significant for *p* < 0.05 (*p* < 0.05 *; *p* < 0.01 **; *p* < 0.001 ***; *p* < 0.0001 ****).

## 3. Results and Discussion

**Figure 2:**
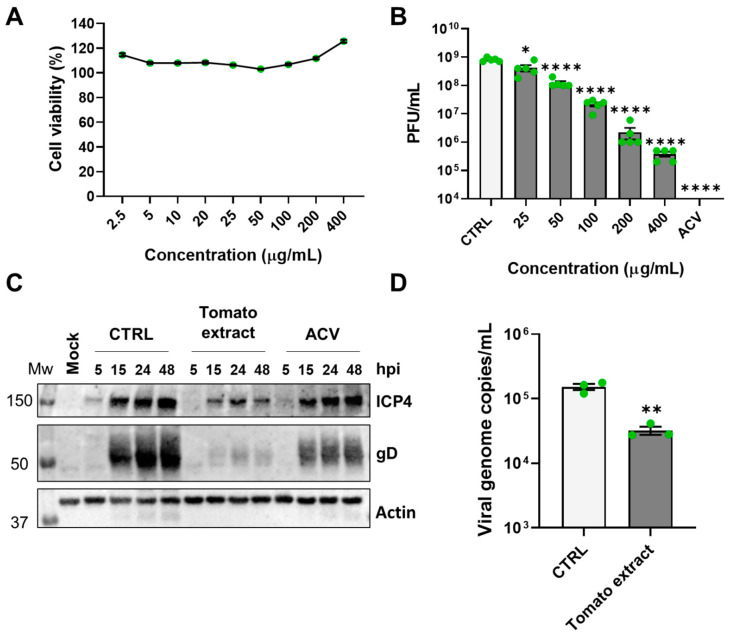
Antiviral activity of tomato extract against HSV-1. (**A**) VERO cells were treated with different concentrations of the exudate or the same amount of vehicle solution (CTRL). Forty-eight hours after treatment, cell viability was determined using the MTT assay. Data are expressed as mean value percent cell viability vs. vehicle control cells (set at 100%). Bars represent the means ± SEM from three independent experiments. (**B**) VERO cells were pre-treated with serial concentrations of tomato extract or CTRL for 1 h. Subsequently, cells were infected with HSV-1 (MOI 0.1), and after the virus adsorption, the viral inoculum was removed; cultures were exposed to the extract or ACV during the infection and for 48 h thereafter. The extent of HSV-1 replication was then assessed by titrating the infectivity of the cell extracts and supernatants combined using a standard plaque assay. Histograms were obtained by plotting the mean plaque counts for each dilution expressed as PFU/mL. Bars represent the means and ± SEM from five independent experiments (*p* < 0.05 *, *p* < 0.0001 ****, unpaired *t*-test tomato extract vs. CTRL). (**C**) VERO cells were treated with the exudate (400 µg/mL) or with an equal volume of CTRL 1 h before infection and for the entire duration of the infection and infected with HSV-1 at an MOI of 1. Samples were collected at the indicated time points, lysed, and subjected to Western blot analyses. ACV was used as a reference drug. Membranes were stained with the indicated antibodies. (**D**) VERO cells were treated with the tomato extract (400 µg/mL) or with the same volume of CTRL and infected with HSV-1 (MOI 1). After 24 h, DNA was extracted from infected cells, and the number of HSV-1 genomes was quantified by qPCR. To determine the number of viral DNA genomes, the primers were used to amplify a segment of the gE gene, and the cellular housekeeping gene GAPDH was used to normalize viral genome counts. Bars represent the means ± SEM from three independent experiments (*p* < 0.01 **, unpaired *t*-test tomato extract vs. CTRL).

In this work, we aimed to study the antiherpetic activity of a root exudate of *Solanum lycopersicum* in vitro (named tomato extract). Initially, we focused on HSV-1, an alpha-herpesvirus highly transmissible and widespread in the population [32,33]. First, the organic extract was prepared from the root exudates of the plants, as described in the materials and methods section. Subsequently, its impact on cellular viability was assessed using a standard MTT assay on uninfected VERO cells, treated with increasing extract concentrations (10–400 µg/mL) or an equal volume of control vehicle (CTRL, DMSO/H_2_O mixture 1:1). The results, depicted in Figure 2A, revealed that the half-maximal cytotoxic concentration (CC_50_) after 48 h of treatment exceeded 400 µg/mL, indicating a lack of significant cytotoxicity at the tested concentrations. To evaluate the potential antiviral activity of the tomato root exudate, we first performed a virus yield reduction assay by treating VERO cells in the presence of serial dilutions of the exudate before, during, and after viral infection to generate a dose–response curve. The extent of HSV-1 replication was then assessed by titrating the infectivity of both supernatants and cell-associated viruses combined through a standard plaque assay in VERO cells. The results presented in Figure 2B demonstrated that the exudate was endowed with anti-HSV-1 activity with the half-maximal inhibitory concentration (EC_50_) value of 25.57 (±8.51) µg/mL. Based on the above results, the SI for HSV-1 is >15.64. Noteworthily, at the highest non-toxic concentration of tomato extract (400 µg/mL), the infectious virus titer was reduced to ~10^5^ PFU/mL, which corresponds to a reduction of over 99% when compared to the vehicle control. Therefore, this concentration was employed in the subsequent experiments. The widely employed antiviral drug ACV was included as a positive control (44.4 µM [34]). As expected, its presence resulted in a complete reduction in viral replication (Figure 2B). To further confirm the antiviral activity, we examined the impact of the treatment on viral protein expression by Western blot analysis. Immunoblotting with specific antibodies was performed to analyze the expression patterns of viral proteins, particularly immediate early/early (ICP4) and late (gD) viral products. A noticeable decrease in viral protein levels was observed at each time point in cells treated with tomato exudate or ACV (Figure 2C). These results suggest that the root exudate interferes with a molecular event that occurs in the early stages of the HSV-1 replication cycle. To further analyze the impact of the tomato exudate on viral DNA replication and the production of new viral genomes, we performed a quantitative real-time PCR (qPCR) analysis. As depicted in Figure 2D, a significant reduction in intracellular copies of the HSV-1 genome was observed after 24 h of treatment, providing additional evidence for the inhibitory impact of the root exudate on viral replication.

**Figure 3:**
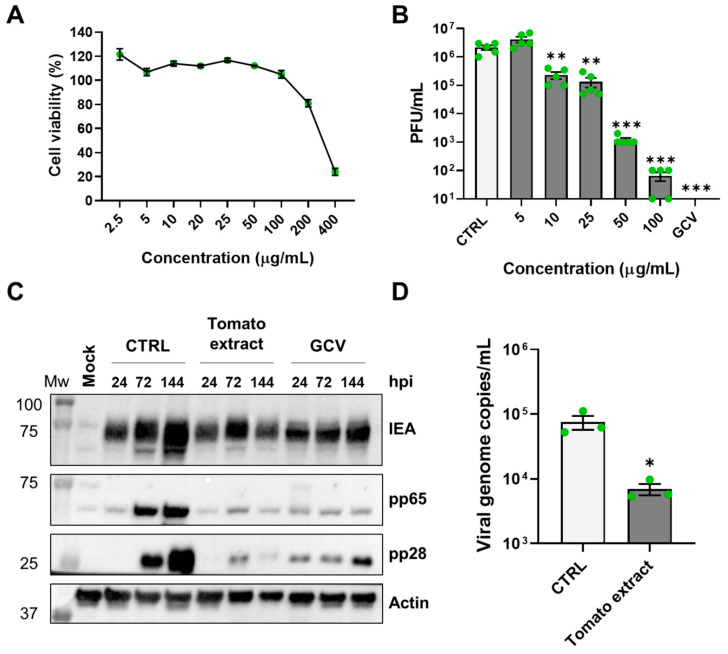
Antiviral activity of the tomato extract against HCMV. (**A**) HFF cells were treated with different concentrations of the exudate or with the same amount of vehicle solution (CTRL). After six days, cell viability was determined using the MTT assay. Data are expressed as mean value percent cell viability vs. CTRL (set at 100%). Bars represent the means ± SEM from three independent experiments. (**B**) HFF cells were pre-treated with serial concentrations of tomato extract for 1 h. Subsequently, cells were infected with HCMV (MOI 0.1), and after the virus adsorption, the viral inoculum was removed; cultures were exposed to the extract, GCV, or CTRL during the infection and for 144 h thereafter. The extent of HCMV replication was then assessed by titrating the infectivity of cell extract and supernatants combined using a standard plaque assay. Histograms were obtained by plotting the mean plaque counts for each dilution expressed as PFU/mL. Bars represent the means ± SEM from five independent experiments (*p* < 0.01 **, *p* < 0.001 ***, unpaired *t*-test tomato extract vs. CTRL). (**C**) HFF cells were treated with tomato extract (100 µg/mL) or with an equal volume of vehicle 1 h before infection and for the entire duration of the infection and infected with HCMV at an MOI of 1. Samples were collected at the indicated time points, lysed, and subjected to Western blot analyses. GCV was used as a reference drug. Membranes were stained with the indicated antibodies. (**D**) HFF cells were treated with the root extract (100 µg/mL) or with the same volume of CTRL and infected with HCMV (MOI 1). After 72 h, DNA was extracted, and the number of HCMV genomes was quantified by qPCR. To determine the number of viral DNA genomes, the primers were used to amplify a segment of the IE1 gene, and the cellular housekeeping gene GAPDH was used to normalize viral genome counts. Bars represent the means ± SEM from three independent experiments (*p* < 0.05 *, unpaired *t*-test tomato extract vs. CTRL).

To determine whether the antiviral activity of *Solanum lycopersicum* was limited to HSV-1 or encompassed other herpesviruses, we investigated its efficacy on the replication of HCMV, which belongs to the *Betaherpesvirinae* subfamily (Figure 3). An MTT viability assay was conducted on HFF cells after 144 h of treatment to establish the CC_50_ dose, which was calculated to be 296.00 (±80) µg/mL (Figure 3A). Through a virus yield reduction assay, performed with serial dilutions of root exudate, we confirmed the antiviral activity of the compound also against HCMV, with an EC_50_ value of 9.17 (±0.65) µg/mL (Figure 3B). The SI for HCMV is 32.28. GCV (25 µM [35]) was included as a positive control.

Similarly to the approach taken for HSV-1, cells underwent Western blot and qPCR analysis after exposure to the extract at concentrations of 100 µg/mL (highly effective non-toxic concentrations, over 99% inhibition). Western blot analysis revealed a decrease in viral protein expression, including immediate early (IEA), early–late (pp65), and late (pp28) proteins, suggesting an overall inhibitory effect on HCMV replication (Figure 3C). Finally, we assessed the extent of intracellular viral genome replication upon treatment with the extract by a qPCR analysis at 72 hpi. Consistently, the results shown in Figure 3D highlight a significant reduction in the viral genome copy number compared to vehicle-treated samples.

**Figure 4:**
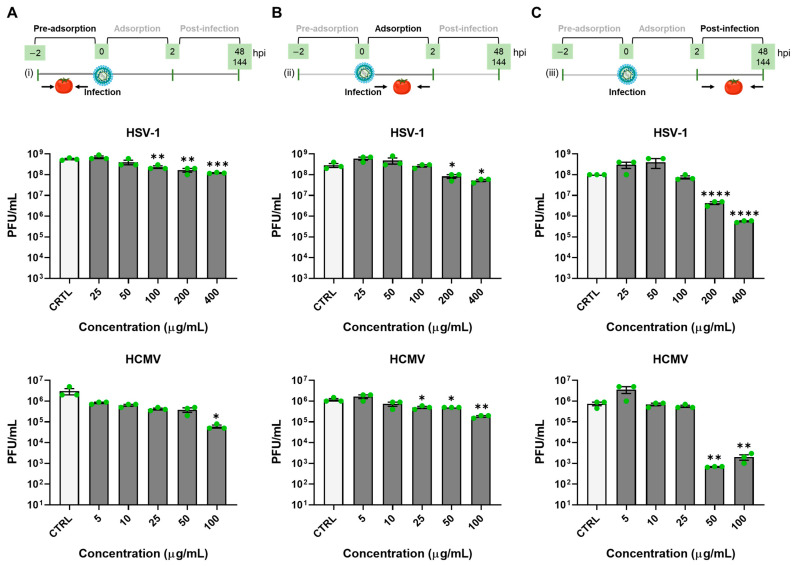
Time of addition assay. VERO or HFF cells were (**A**) pretreated with serial dilutions of tomato extract for 1 h, after which, treatments were removed and cells were infected with HSV-1 (VERO) or HCMV (HFF) (MOI 0.1); (**B**) infected and treated simultaneously for 2 h, after which treatments and viral inoculum were removed; (**C**) infected as described above and, after the removal of inoculum, treated with increasing concentrations of the compound. For all the conditions, cells were incubated for 48 h (VERO) or 144 h (HFF); then, samples were harvested, and infectivity titers were determined by a standard plaque assay. Histograms were obtained by plotting the mean plaque counts for each dilution expressed as PFU/mL. Bars represent the means ± SEM from three independent experiments (*p* < 0.05 *, *p* < 0.01 **, *p* < 0.001 ***, *p* < 0.0001 ****, unpaired *t*-test tomato extract vs. CTRL).

Next, to identify which phase of the viral replication cycle was mainly affected by the treatment, serial dilutions of extract were added to VERO or HFF cells at different time schedules: (i) one hour before infection (pre-adsorption stage), (ii) for 2 h during viral adsorption and then removed (adsorption stage), (iii) after the removal of viral inoculum (post-adsorption stage). For each condition, the infected samples were collected at 48 hpi for HSV-1 and 144 hpi for HCMV. Plaque assay titration revealed that the most substantial antiviral effect occurred when the root extract was added after the infection (Figure 4C), compared to the pre-adsorption (Figure 4A) or the adsorption (Figure 4B) conditions.

Our results align with prior research that underscores the use of various Solanum-genus plants, including *Solanum dulcamara*, *Solanum lyratum*, and *Solanum nigrum*, as traditional anti-herpes agents since ancient times [36]. Importantly, our study extends this activity for the first time to HCMV, representing another member of the *Herpesviridae* family. Studies indicate that Solanum steroidal glycosides, particularly spirostanol glycosides, inhibit HSV-1. It has been suggested that the activity is influenced by the type of oligosaccharide moiety, but the underlying mechanism remains unclear [36]. Additionally, extracts from *S. paniculatum* leaves demonstrated efficacy against HSV-1 in a VERO cell in vitro model, albeit with a lower SI compared to our product. In this context, the mechanism of action has not been elucidated [37]. Notably, *Solanaceae* glycoalkaloids insert glycones into the viral envelope, causing HSV-1 virion leakage [26]. While we cannot completely rule out a similar mechanism for root exudate extracts, our observations suggest only minimal inhibition when the compound is added during viral pre-adsorption and adsorption (i.e., attachment and entry phases). Since the primary inhibitory effect of the extract was prominently observed when added post-infection, we can infer that the root extract interferes with a molecular event during the herpesvirus replication stage. These findings are consistent with viral protein expression and qPCR results (Figure 2 and Figure 3, panels C and D) and align with earlier studies on DENV [24] and CHIKV [25] viral models.

**Figure 5:**
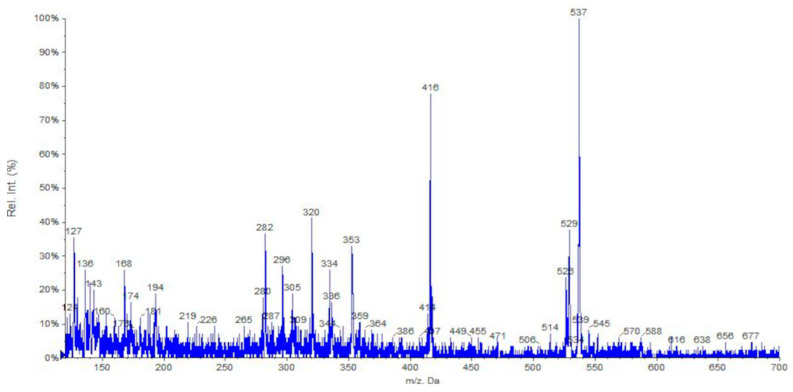
Full-scan positive ion ESI-MS spectrum of a solution of tomato root exudate. Solution infused: 1.0 mg/mL in acetonitrile + 0.1% formic acid.

Finally, the phytochemical composition of the *Solanum lycopersicum* root exudate was investigated through mass spectrometry. Specifically, electrospray ionization (ESI-MS) was employed, enabling the acquisition of mass spectra with a low degree of fragmentation and the qualitative characterization of the complex mixture, consisting of small chemicals and biomolecular components [38]. The full-scan positive ion ESI-MS spectrum is depicted in Figure 5, with detailed information on the major components presented in Table 1.

Five main classes of biologically derived compounds were identified in the ESI-MS spectrum belonging to different substances (see Table 1) commonly found in tomato extracts: carotenoids, glycoalkaloids, phytosterols, flavonoids, and amino acids [39,40]. The first class, carotenoids, includes the typical fragment of ionized tetraterpene derivatives lycopene and β-carotene (M.W. 536.88 g/mol, *m*/*z* = 537 [M + H]^+^), while doubly charged adducts can be found for lutein (M.W. 568.87 g/mol, *m*/*z* = 296 [M + H + Na]^2+^), phytoene (M.W. 544.95 g/mol, *m*/*z* = 282 [M + H + NH_4_]^2+^), and phytofluene (M.W. 542.94 g/mol, *m*/*z* = 280 [M + H + NH_4_]^2+^). Glycoalkaloid derivatives identified were α-tomatine, with the molecular fragment at *m*/*z* = 529 which might result from the formation of a doubly charged adduct (M.W. 1034.18 g/mol, [M + H + Na]^2+^), its corresponding aglycone tomatidine (M.W. 415.66 g/mol, *m*/*z* = 416 [M + H]^+^), and lycoperoside H (M.W. 1049.54 g/mol, *m*/*z* = 525 [M + 2H]^2+^). The ionized adduct at *m*/*z* = 416 can be assigned to the phytosterol derivative β-sitosterol (M.W. 414.72 g/mol, [M + H]^+^). The fourth class of polyphenolic compounds (flavonoids) identified in the tomato root exudate includes myricetin (M.W. 318.04 g/mol, *m*/*z* = 336 [M + NH_4_]^+^ and *m*/*z* = 168 [M + H + NH_4_]^2+^), isorhamnetin (M.W. 316.26 g/mol, *m*/*z* = 334 [M + NH_4_]^+^), quercetin (M.W. 302.24 g/mol, *m*/*z* = 320 [M + NH_4_]^+^), and kaempferol (M.W. 286.23 g/mol, *m*/*z* = 287 [M + H]^+^). Lastly (glyco)amino acid derivatives *N*-acetylglucosamine-asparagine (Glc*N*Ac-(1→*N*)-Asn, M.W. 335.31 g/mol, *m*/*z* = 353 [M + NH_4_]^+^), *N*-acetyl-L-hydroxyproline (AHYP, M.W. 173.17 g/mol, *m*/*z* = 174 [M + H]^+^), and homocysteine (M.W. 135.18 g/mol, *m*/*z* = 136 [M + H]^+^) were identified.

The results of this qualitative analysis show that the root exudate of *Solanum lycopersicum* contains a number of phytochemicals with recognized antiviral activity. In particular, some key identified phytochemicals in the root exudate possess antiherpetic properties, as demonstrated for lycopene (EC_50_ = 22.86 μg/mL for HSV-1) [41], β-sitosterol (EC_50_ = 2.7 μg/mL for HSV-2) [42], tomatidine [26], and several flavonoids [43,44], including quercetin (EC_50_ = 52.90 μg/mL for HSV-1 and EC_50_ = 70.01 μg/mL for HSV-2) [45,46], kaempferol [47], and myricetin [44]. Furthermore, natural antioxidants, such as bioactive carotenoids (lycopene, β-carotene, lutein, phytoene, phytofluene), phytosterols (β-sitosterol), and flavonoids (quercetin, kaempferol, myricetin,) have a beneficial role in human health and chronic diseases owing to their ability to modulate the activity of specific enzymes or inhibit the production of reactive oxygen species (ROS) acting as free radical scavengers [48,49,50]. Additionally, it is well known that the pharmacological profile of flavonoids, carotenoids, glycoalkaloids, and phytosterols includes anticancer, anti-inflammatory, anti-allergenic, antithrombotic, antimicrobial, antioxidant, vasodilator, and cardioprotective effects [51,52,53,54,55].

Since phytoextracts are mixtures of secondary metabolites, the pharmacological behavior of plant extracts is often due to the synergic effect of two or more active constituents rather than a single component. Indeed, the results presented in this work can be rationalized assuming that the biologically active compounds detected in the *Solanum lycopersicum* root exudate might play a synergic role in the antiviral activity against herpesviruses. The combination of the antiherpetic activity of the detected phytochemicals with their antioxidant properties and other pharmacological profiles make this extract a promising candidate for the development of the root exudate of *Solanum lycopersicum* as a new combined phytomedicine. Overall, these findings could potentially contribute to progress in the field of antiviral phytotherapy, which has experienced a resurgent interest in the field of antiviral drug discovery owing to the increasing urge to contest the development of drug resistance [56].

## 4. Conclusions

In conclusion, our research highlights the potential of tomato plants as a natural product exhibiting antiviral activity against herpesviruses. The exudate from *Solanum lycopersicum* root significantly inhibited HSV-1 and HCMV replication in VERO and HFF cells, with EC_50_ values of 25.57 and 9.17 µM, respectively. The SIs of >15.64 for HSV-1 and 32.28 for HCMV imply a substantial therapeutic window, indicating that the drug can effectively inhibit the target while maintaining a relatively low cytotoxic impact on host cells, making it a promising candidate for further preclinical and clinical development. Subsequent protein expression analysis and time-of-addition experiments revealed that the antiviral effect is mainly produced in post-infection conditions, during the viral replication phase, as no or a limited antiviral effect was detectable for the pre-infection and during-infection treatments. These findings suggest that *Solanum lycopersicum* could be a promising alternative to current antiviral drugs. Further investigation is necessary to understand the specific mechanisms of action, the efficacy in vivo, and the pharmacokinetics of the extract. Finally, it will be crucial to identify the more active molecules within the root exudate responsible for the observed antiviral effects. Nevertheless, this study offers valuable insights into the potential of *Solanum lycopersicum* as a source of natural antiviral agents in the ongoing efforts to combat viral infections and uphold public health safety.

## Figures and Tables

**Table 1:** Identification of phytochemicals of tomato root exudate components. Analytically significant ions (*m*/*z*) are reported.

Compound	Molecular Formula	M.W. (g/mol)	Ionized Adduct	Signal (*m*/*z*)
Lycopene	C_40_H_56_	536.88	[M + H]^+^	537
β-Carotene	C_40_H_56_	536.88	[M + H]^+^	537
α-Tomatine	C_50_H_83_NO_21_	1034.18	[M + H + Na]^2+^	529
Lycoperoside H	C_50_H_83_NO_22_	1049.54	[M + 2H]^2+^	525
β-Sitosterol	C_29_H_50_O	414.72	[M + H]^+^	416
Tomatidine	C_27_H_45_NO_2_	415.66	[M + H]^+^	416
Glc*N*Ac-(1→*N*)-Asn	C_12_H_21_N_3_O_8_	335.31	[M + NH_4_]^+^	353
Myricetin	C_15_H_10_O_8_	318.04	[M + NH_4_]^+^ [M + H + NH_4_]^2+^	336 ^a^ 168
Isorhamnetin	C_16_H_12_O_7_	316.26	[M + NH_4_]^+^	334
Quercetin	C_15_H_10_O_7_	302.24	[M + NH_4_]^+^	320
Lutein	C_40_H_56_O_2_	568.87	[M + H + Na]^2+^	296
Kaempferol	C_15_H_10_O_6_	286.23	[M + H]^+^	287 ^a^
Phytoene	C_40_H_64_	544.95	[M + H + NH_4_]^2+^	282
Phytofluene	C_40_H_62_	542.94	[M + H + NH_4_]^2+^	280 ^a^
AHYP	C_7_H_11_NO_4_	173.17	[M + H]^+^	174 ^a^
Homocysteine	C_4_H_9_NO_2_S	135.18	[M + H]^+^	136

^a^ Low-abundance ions.

## Data Availability

Data are contained within the article.
